# Neural responses to category ambiguous words

**DOI:** 10.1016/j.neuropsychologia.2015.01.036

**Published:** 2015-01-28

**Authors:** Erin Conwell

**Affiliations:** Erin Conwell, North Dakota State University, NDSU Dept. 2765, P.O. Box 6050, Fargo, ND 58108-6050, 1-701-231-6123, Erin.R.Conwell@ndsu.edu

**Keywords:** Event related potential, lexical category ambiguity, prosody, lexical representation

## Abstract

Category ambiguous words (like hug and swing) have the potential to complicate both learning and processing of language. However, uses of such words may be disambiguated by acoustic differences that depend on the category of use. This article uses an event-related potential (ERP) technique to ask whether adult native speakers of English show neural sensitivity to those differences. The results indicate that noun and verb tokens of ambiguous words produce differences in the amplitude of the ERP response over left anterior sites as early as 100 ms following stimulus onset and persisting for over 400 ms. Nonsense words extracted from noun and verb contexts do not show such differences. These findings suggest that the acoustic differences between noun and verb tokens of ambiguous words are perceived and processed by adults and may be part of the lexical representation of the word.

## 1. Introduction

Like many languages, English contains words that may be used in more than one lexical category (e.g., noun/verb homophones like run and fence). These words can produce temporary ambiguities when they are used in sentences and could, in principle, cause significant problems for learners who are trying to sort the words they hear into appropriate lexical categories. However, some research suggests that these words, although homophonous at the segmental level, may contain acoustic cues that differentiate their uses (Conwell & Morgan, 2012; Shi & Moisan, 2008; Sorensen, Cooper & Paccia, 1978) and that infants are sensitive to those cues (Conwell & Morgan, 2012). Whether adults are similarly sensitive, however, is an open question. Infants show greater sensitivity to a wider range of phonetic distinctions than adults do (Werker & Tees, 1999), so although adults produce noun and verb tokens of homophones differently, they may not perceive those differences. This article examines whether adult English speakers show neural discrimination of isolated tokens of noun/verb homophones.

### 1.1 Nouns, verbs and category ambiguity

Instead of using semantically-driven elementary school definitions such as “a noun is a person, place or thing,” linguists categorize words based on their grammatical properties. Nouns are words with noun-like syntax and morphology. They may be the subjects of sentences or the objects of verbs and prepositions. Verbs are words with verb-like syntax and morphology, taking noun phrases and prepositional phrases as arguments. These functional definitions are inherently circular, as “verb-like” syntax requires a definition of “noun” and “noun-like” syntax requires a definition of “verb.” Several researchers have proposed methods of “distributional bootstrapping” that children might use to break into this system (Maratsos & Chalkley, 1980; Mintz, 2003; Monaghan, Chater & Christiansen, 2005). These proposals differ in the details, but in broad terms, they consider whether co-occurrence patterns of nouns and verbs with distinct, highly frequent function words might allow children to create ersatz categories that contain mostly nouns and mostly verbs. Under some implemented models of distributional bootstrapping (e.g., Mintz, 2003), these small categories containing mostly nouns and mostly verbs would be combined on the basis of overlap in items. Noun/verb homophones could confound this process, as a word such as run could reasonably appear in both noun and verb contexts. For this reason, these words have been used to argue against the very possibility of distributional bootstrapping (e.g., Pinker, 1987).

Recent developmental research indicates, however, that this problem may not be as significant as it has been made out to be. Parents produce acoustic distinctions between noun and verb uses of both real and novel words when speaking to children (Conwell & Morgan, 2012; Shi & Moisan, 2008) and infants are sensitive to these differences (Conwell & Morgan, 2012). This suggests that distributional bootstrapping need not fall victim to noun/verb homophone confusion because infants may be able to maintain two distinct lexical entries for such words, one that is a noun and one that is a verb. If this were the case, infants would not conflate noun and verb categories because noun tokens of homophones would not be considered “the same” as their verb counterparts.

Unanswered in this previous work is the question of whether children maintain sensitivity to these distinctions as they age and whether these distinctions might be incorporated into their representations of the words. In other words, if infants are sensitive to acoustic distinctions between noun and verb uses of homophones, do they establish lexical representations that remain distinct as they develop? Perceptual narrowing of phonetic categories is well documented in the literature on speech perception. For example, children show reduced sensitivity to non-native consonant contrasts around 10–12 months of age (Werker & Tees, 1999). However, the children in the Conwell & Morgan (2012) study were 13 months old and still sensitive to prosodic differences in noun/verb homophone pairs, indicating that these differences continue to be perceived even after sensitivity to some non-native phonemic contrasts has weakened. It is possible, therefore, that perceptual sensitivity to these distinctions is preserved across development.

The fact that adults reliably produce these differences may be uninformative regarding their status as part of the lemma or, for that matter, adults’ perceptual sensitivity to such information. Adults reliably produce allophonic variations that are conditioned by context, but show reduced sensitivity to the distinctions between those allophones (e.g., aspiration of stop consonants by English speakers). Therefore, there are two possibilities regarding the production of acoustic distinctions in noun/verb homophones by adults. First, these distinctions may arise solely as the result of prosodic processes in production. In that case, adults’ representations of noun/verb homophones may consist of only one form and we would not expect adults to be sensitive to this variation. Alternatively, these distinctions, although a by-product of prosody, may be attached to the lemma itself during the learning process, in which case adults should show preserved sensitivity to them.

### 1.2 Use of prosodic information in development

The distinctions that have been observed between noun and verb uses of homophones may arise because of an interaction between sentence-level prosody and the usual distributions of nouns and verbs in sentences. Specifically, noun tokens tend to be longer than verb tokens of the same word (Conwell & Barta, under revision; Conwell & Morgan, 2012; Sorensen, et al., 1978) and nouns are more likely than verbs to appear in phrase-final position in sentences. English has robust phrase-final lengthening (Shattuck-Hufnagel & Turk, 1996), which would explain why words that are more likely to be at the ends of phrases are also more likely to be longer in duration.

Prosodic cues are used to facilitate sentence processing across the lifespan. Infants use phrase-final and sentence-final prosody to bundle words, preferring to listen to phrases that were prosodically coherent during a familiarization period (Nazzi, Kemler Nelson, Jusczyk & Jusczyk, 2000; Soderstrom, Seidl, Kemler Nelson & Jusczyk, 2003). Adults can use prosodic information to resolve syntactic ambiguities (Kjelgaard & Speer, 1999) and word-level prosody, such as syllabic stress, distinguishes meanings of some words in English (e.g., inCENSE and INcense; Sereno & Jongman, 1995). Preschool-aged children also use prosody to disambiguate sentence structure, although these effects are slower in children than in adults (Snedeker & Yuan, 2008) and there is some evidence that children fail to use focal stress in an adult-like way when processing sentences (Cutler & Swinney, 1987). Most studies, however, do not ask whether prosody affects the processing of individual monosyllabic words, as the research on homophone perception by infants suggests.

There are two ways that prosody could affect interpretation of words. The first is that prosodic regularities (e.g., noun uses tend to be longer than verb uses) are encoded in the lemma itself. That is to say, homophones are homophonous at the segmental level, but meanings are linked to word forms that are suprasegmentally distinct. Prior evidence indicates that less frequent meanings of homophones are longer in duration than tokens that capture more frequent meanings (e.g., thyme is longer in duration than time; Gahl, 2008). Adults also use emotional prosody to disambiguate senses of homophones with distinct emotional valence (e.g., bridal and bridle; Nygaard, Patel & Queen, 2002). Likewise, meanings that are distinct in their syntactic properties (i.e., noun/verb homophones) could be suprasegmentally distinct, not just in production, but in their representation. Alternatively, the distinct meanings of homophones could be linked to a single phonological form, but prosody could function like referential context to disambiguate the meanings. Under this account, prosodic cues to the lexical category of an ambiguous word arise as by-products of syntax and are not inherent in the representation of that word.

The research on infants’ ability to distinguish between noun and verb uses of homophones does not differentiate these accounts (Conwell & Morgan, 2012). That study showed only that infants can perceive the distinction, not that it is incorporated into their representations of the words. If the prosodic distinctions are part of the representation itself, then adults should show effects associated with the particular lexical category of use. In other words, verb uses of noun/verb homophones should elicit different responses from adults than noun uses do. Translating infant methods for use with adult participants is challenging for a range of reasons, but neural methods allow for implicit responses to stimuli in a way that many behavioral methods typically used with adults do not.

### 1.3 ERP responses to nouns and verbs

Research using event related potentials (ERPs) measured with electroencephalography (EEG) provides a means of measuring neural response to stimuli. In the domain of language research, ERPs have been used to examine lexical access using both visual and auditory stimuli. Brown and colleagues (Brown, Marsh & Smith, 1973; 1976; 1979) exposed participants to noun/verb homophones in sentence contexts while recording from 4 electrodes on the scalp. In one of these studies, participants heard identical auditory tokens of these words spliced into carrier phrases that produced either a noun or verb interpretation (Brown, et al., 1973). Brown and colleagues report a differential response to noun and verb uses in an early negative-going component for the left anterior, but not the right anterior, electrode. In later research, Brown and colleagues (1976; 1979) presented the tokens in ambiguous phrases and instructed participants to interpret those phrases with either a noun or a verb reading at the beginning of each epoch. In this case, participants again showed discrimination at the left anterior recording site both at 150 ms after stimulus onset and in a later period between 390 and 500 ms. These findings suggest that neural responses to noun/verb homophones differ depending on the interpretation of those words by the participant.

Work examining the processing of unambiguous nouns and verbs shows that frontal sites produce differences in the amplitude of a negative-going component beginning around 250 ms following stimulus onset and continuing for another 250 ms (Molfese, Burger-Judisch, Gill, Golinkoff & Hirsh-Pasek, 1996). Specifically, nouns produced greater amplitude than verbs did. Research with preschool-aged children likewise finds amplitude differences in a negative component in the same time frame, although in this case, verbs produced greater amplitude than nouns did (Tan & Molfese, 2009). These studies use auditory presentation of words with matching or mismatching videos. Using visual presentation of text similarly produces differences in response to unambiguous nouns and verbs around 230 ms following stimulus onset, with verbs eliciting greater negativity than nouns (Pulvermüller, Lutzenberger & Preissl, 1999). Because the stimuli used in these studies were all unambiguous nouns and verbs, it is unclear whether these effects arise because of the lexical category of the word, per se, or because nouns typically have more concrete, visualizable referents, while verbs refer to actions (Kellenbach, Wijers, Hovius, Mulder & Mulder, 2002).

Federmeier, Segal, Lombrozo & Kutas (2000) presented noun/verb ambiguous words and unambiguous nouns and verbs visually while recording EEG responses. The ambiguous words were presented in disambiguating sentence contexts. They find that ambiguous words produce a greater negativity over frontal regions than unambiguous words do. Furthermore, uses of ambiguous words in noun contexts produce a more negative response over frontal sites than uses in a verb context. These findings indicate that the grammatical context in which the word is used can elicit differences in the ERPs. However, like the research by Brown and colleagues (1973; 1976), this work uses sentence context to indicate whether the word is being used as a noun or a verb. There is, to date, no evidence regarding the processing of isolated auditory tokens of noun/verb homophones. If adults differentiate and assign category membership to isolated noun/verb homophones based on the acoustic properties of these words, we might expect differences in the ERP response to such tokens. Specifically, we would expect the same kinds of differences that are found when syntax disambiguates category.

This article presents results from an ERP study asking whether prosodic distinctions between noun and verb uses of noun/verb homophones are part of the representations of those words. If they are, isolated tokens of noun/verb homophones should elicit different neural responses depending on the category of use. The same effects should not be found for nonsense words extracted from identical noun and verb contexts, as those words do not have a representational status. Based on previous findings on neural processing of nouns and verbs (e.g., Brown, et al., 1973; 1976), we would predict differences in the ERP to ambiguous nouns and verbs over left frontal sites, but no differences between nonsense words. Such findings suggest that the perceptual effects previously reported in infants (Conwell & Morgan, 2012) persist into adulthood, demonstrating that prosodic distinctions between noun and verb uses of homophones are maintained over development. This would support the possibility that such information is part of the lemma, rather than a mere by-product of prosodic processes.

## 2. Method

If noun and verb uses of homophones are represented distinctly, they should evoke different neural responses. To test whether neural distinctions between noun and verb tokens might present a clearer picture of how adults process these words, we now examine whether ERPs measured by EEG are distinct for noun and verb uses of homophones.

### 2.1 Participants

The participants in this study were 17 right-handed native speakers of English (7 male and 10 female) recruited from the NDSU community. Participants were between 18 and 33 years old and reported normal hearing and no history of neurological problems or language disorders. An additional 15 participants completed the study but were excluded from analysis because of left-handedness (2), equipment failure (1) or too few usable trials due to movement artifacts (12)^1^. For inclusion in analysis, participants were required to have a minimum of 48 usable trials (out of a possible 72) in every condition. The participants who were included in the analysis had an average of 64.8 usable trials per condition. Participants received either course credit or small monetary compensation in exchange for their participation.

### 2.2 Stimuli

**Figure 1:**
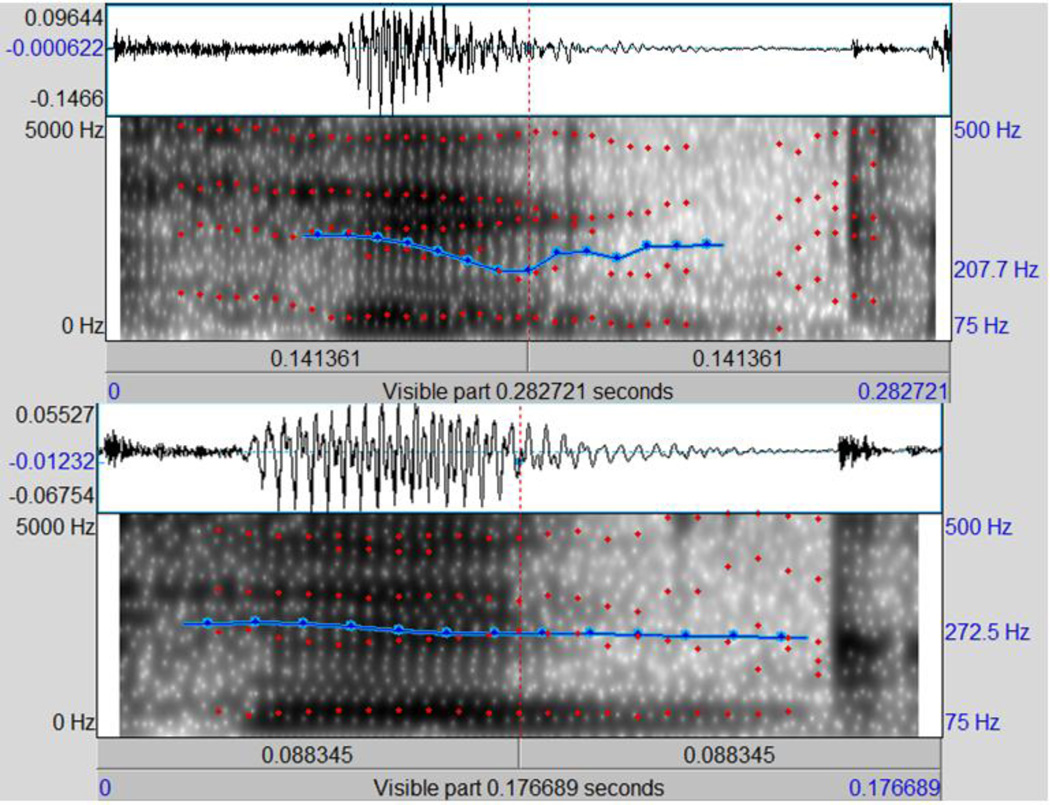
Waveform and spectrogram for a noun token (top) and verb token (bottom) of kick used in this study.

Native English speakers, two male and two female, recorded a set of passages containing tokens of noun/verb homophones in both sentence medial and sentence final positions. Four other native English speakers, two male and two female, recorded the same passages with the noun and verb homophones replaced with non-words. These speakers were all naïve to the purpose of the study. The sentence medial tokens were extracted from these passages and used as the stimuli for this study. Participants heard noun and verb tokens of each word or non-word produced by each speaker, for a total of 288 tokens over the course of the experiment. All stimuli were normalized for average amplitude. Sample waveforms and spectrograms for noun and verb uses of kick are shown in Figure 1.

To determine whether the stimuli contained acoustic differences that might be perceived by participants, all stimuli were measured using the PRAAT program (Boersma & Weenink, 2014) for duration, vowel duration, mean pitch, pitch range and the first and second formants at the midpoint of the vowel. A series of 2×2 (lexical category × lexical status) ANOVAs were conducted for each of these dependent variables. Token duration showed a main effect of lexical status (F(1,287)=5.713, p=.017, partial η^2^=.02) and of lexical category (F(1,287)=3.892, p=.049, partial η^2^=.014). Vowel duration also showed main effects for both lexical status (F(1,287)=20.53, p<.001, partial η^2^=.068) and lexical category (F(1,287)=4.78, p=.03, partial η^2^=.017). For both measures, nouns were longer than verbs and nonsense words were longer than real words. Mean pitch showed a significant main effect of lexical status (F(1, 287)=21.88, p>.001, partial η^2^=.072), with nonce words exhibiting greater average pitch than real words. The analysis of pitch range revealed no main effects of either factor (both p>.42). The first vowel formant showed a significant main effect of lexical status (F(1,287)=9.013, p=.003, partial η^2^=.031) and the second vowel formant showed a marginal main effect of the same factor (F(1,287)=2.996, p=.085, partial η^2^=.01). In both cases, real words had higher formants than the nonce words. None of these dependent variables revealed a significant interaction of lexical category and lexical status (all p>.244).

Planned t-tests indicate that uses of real nouns were marginally longer in duration than verb uses of the same words (t(142)=1.9, p=.06) and contained longer vowels (t(142)=2.89, p=.004). Real noun tokens did not differ from real verb tokens in their average pitch, pitch range or vowel formants. Nonsense noun uses did not differ from nonsense verb uses on any of the measured dimensions (all p>.29).

### 2.3 Procedure

Each participant had the sensor net applied to his or her head and was then led into a darkened, sound-attenuated room. Participants sat approximately 50 cm from a computer monitor and a pair of desktop speakers. An EPrime2 program was used to present the stimuli one at a time over the speakers at a consistent, comfortable volume. To ensure that participants were accessing lexical representations and attending to the stimuli, they were given instructions standard for an auditory lexical decision task. They were to indicate whether each word was a real word or a non-word as quickly and accurately as possible by pressing one of two keys on a button box resting on the desk in front of them. The next stimulus was presented 1000 ms after the participant provided a response. Participants heard each stimulus once for a total of 288 trials, 72 per condition. Presentation of stimuli was fully randomized.

#### 2.3.1 ERP Recording and Analysis

Event related potentials were recorded with a 64-channel Hydrocel Sensor Net v2.0 (Electral Geodesics, Inc., Eugene, OR). EEG was continuously recorded and referenced to the Cz electrode. The EEG signal was amplified via an EGI NetAmps 200 amplifier using a sampling rate of 250 Hz and a bandpass filter of 0.1–100 Hz. Impedances were checked online before each experimental session and the experiment began only when impedances were below 100 kΩ; the EGI system is a high-impedance system that is calibrated for recording at this impedance threshold. Impedances at this level do not significantly affect data quality, except under very humid recording conditions (Kappenman & Luck, 2010).

Each participant’s continuous EEG data were processed following the experimental session using NetStation 4.3.1 (Eugene, OR). Data were lowpass filtered at 30 Hz. The EEG signal was divided into 1000 ms segments that included a 100 ms pre-stimulus baseline period and a 900 ms period following stimulus onset. To ensure that segments were compared to baseline, the average voltage in the baseline period was subtracted from the entire segment. Following this baseline correction, automated routines detected ocular artifacts and removed those artifacts. Each participant’s data was then averaged by condition. All artifact-free trials were included in these averages, regardless of the accuracy of the behavioral response. Data from each participant was then re-referenced to an average reference. When sampling from a large number of electrodes distributed over the entire scalp, the average reference provides a good estimate of the actual voltage across the head (Picton, et al., 2000).

**Figure 2:**
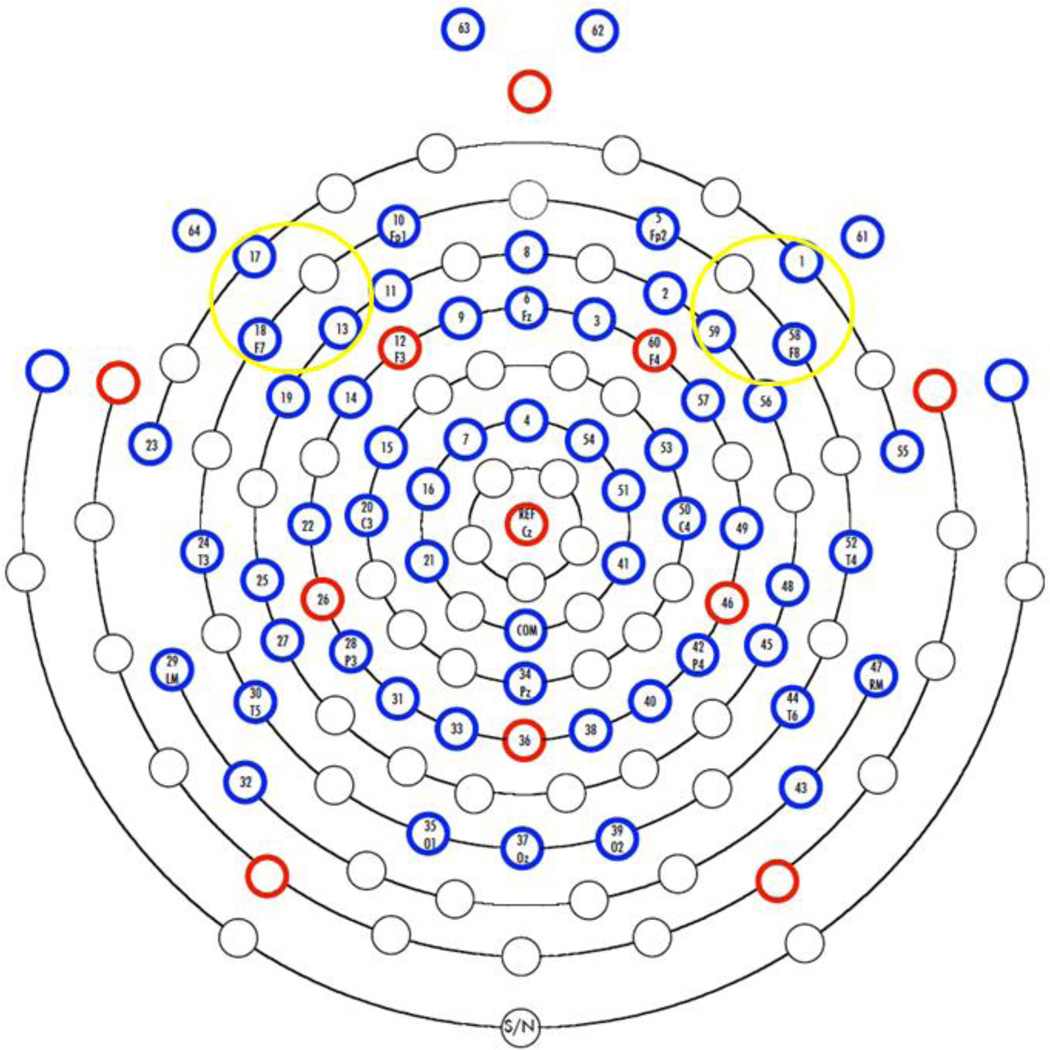
The layout of the sensor net. Sensors included in the analysis are circled in yellow.

Three electrodes per hemisphere were selected as channels of interest for analysis. These channels were selected on the basis of locations reporting differences in previous research (lateral, anterior; see Brown, et al., 1973) and visual examination of the grand average. These included F5, F7 and F9 (left) and F6, F8 and F10 (right). The electrodes used in the analysis are highlighted in Figure 2. The data from these electrodes were averaged over each hemisphere. Three time windows were selected for analysis on the basis of previous research (e.g., Molfese, et al., 1996) and visual inspection of the grand average. These included two early components at 70–160 ms post-stimulus onset (P1) and 170–270 ms (N1), as well as a broad late component ranging from 275–525 ms. Because this study examined effects at frontal, lateralized sites, this last component is not taken to be analogous to the N400, which is typically broadly distributed, although it covers approximately the same time range. The breadth of these components accounts for variability among participants, ensuring that individual variation did not cause some participants’ components to be truncated in the analysis. Analysis was conducted using mean amplitude within the time window for each condition, averaged across the three sensors in each hemisphere. Latency effects were not analyzed because the noun and verb tokens differ primarily in duration and this would be the likely basis of any latency effect. The data were analyzed using repeated-measures ANOVAs.

## 3. Results

### 3.1 Behavioral Results

**Figure 3:**
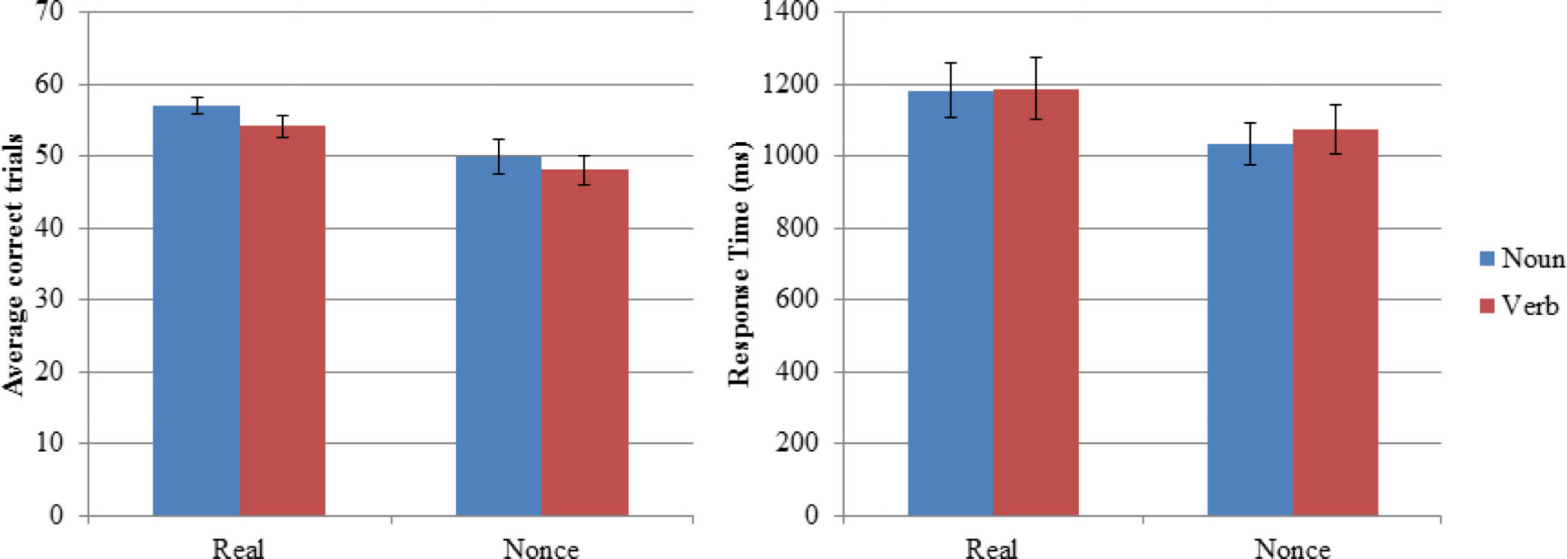
Behavioral data from ERP participants. The chart on the left displays the number of accurate trials per condition and the chart on the right shows mean response times by condition. Error bars represent standard error of the mean.

Accuracy in the lexical decision task was affected by both the lexical status of the word and by the lexical category of the token (Figure 3). A 2×2 repeated-measures ANOVA revealed significant main effects of lexical status (F(1, 16)=5.046, p=.04, partial η^2^=.240) and of lexical category (F(1, 16)=11.98, p=.003, partial η^2^=.428), but no significant interaction of the two (F(1, 16)=.638, p=.421, partial η^2^=.041). Participants more accurately categorized real words (M=55.62) than nonsense words (M=48.67), regardless of lexical category. Noun uses were more accurately categorized (M=53.5) than verb uses (M=51.09), regardless of lexical status.

Average response times are also shown in Figure 3. A 2×2 repeated-measures ANOVA using response times as the dependent variable found a significant main effect of lexical status (F(1,16)=19.25, p<0.001, partial η^2^=.546). Participants were faster to respond to real words (M=1054 ms) than they were to nonsense words (M=1184 ms). The ANOVA revealed no main effect of lexical category (F(1,16)=1.424, p=0.25, partial η^2^=.082) and no lexical category by lexical status interaction (F(1,16)=.556, p=.467, partial η^2^=.034). Average response times across conditions indicate no overlap with the components of interest in the ERP analysis.

### 3.2 ERP Results

**Figure 4:**
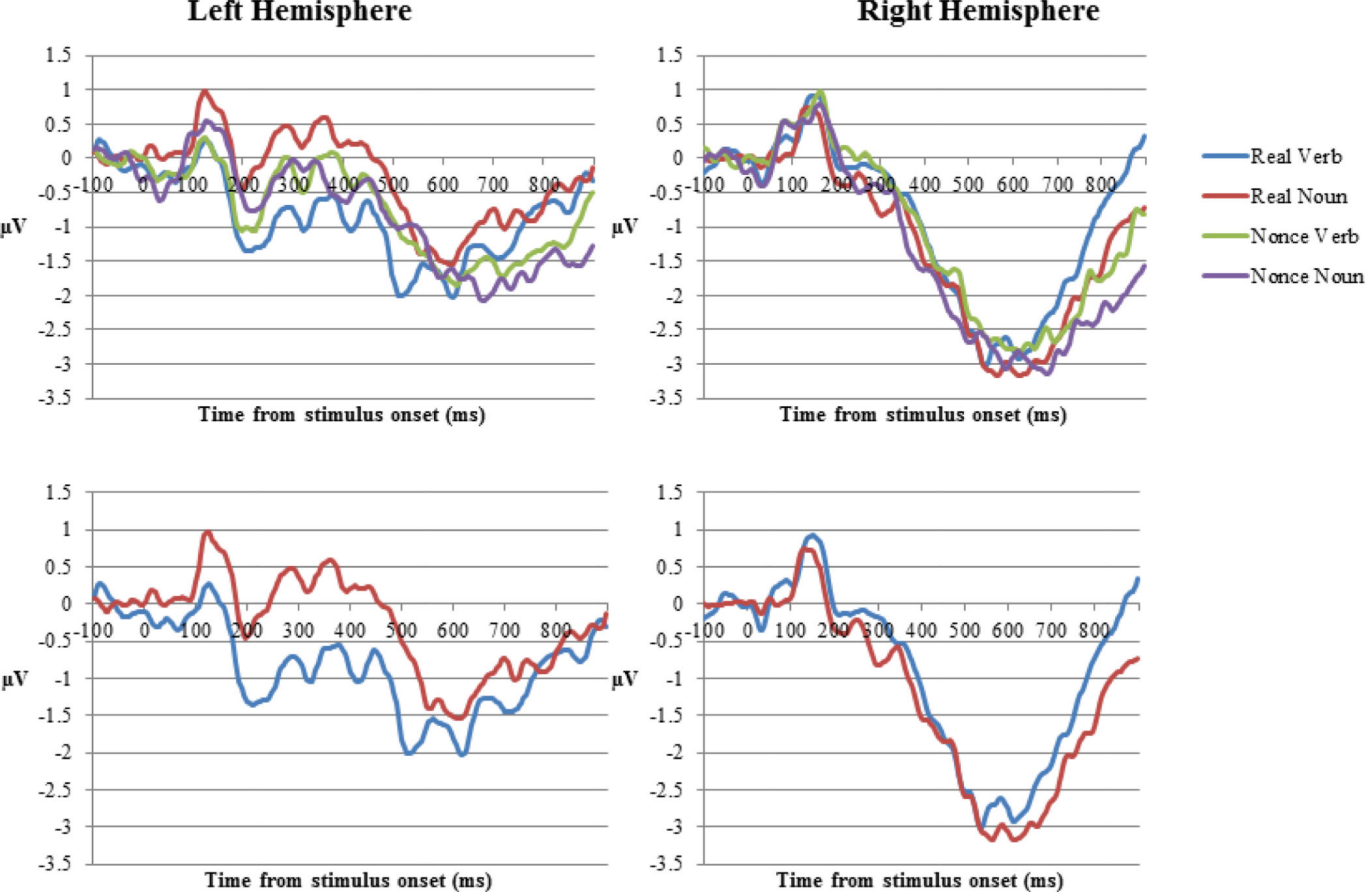
Grand average ERPs measured over left and right lateral anterior sites for real and nonsense noun and verb tokens. The top plots contain data from all conditions, while the lower plots contain responses to only the real nouns and verbs.

All 17 participants exhibited a positive peak around 100 ms post-onset, a negative peak around 200 ms post-onset and a slow component beginning around 275 ms and continuing late into each trial. The average waveform by condition by hemisphere is shown in Figure 4. Mean amplitudes at each of the three time windows of interest were analyzed using a 2×2×2 (lexical category (noun/verb) × lexical status (real/nonce) × hemisphere (left/right)) repeated-measures ANOVA.

#### 3.2.1 P1 Amplitude

Analysis of the mean amplitude during the initial positive peak shows a significant main effect of hemisphere, indicating a lower average amplitude in the left hemisphere than in the right (F(1, 16) = 4.96, p=.041, partial η^2^=.24) and a significant lexical category by hemisphere interaction (F(1, 16)=6.06, p=.026, partial η^2^=.275). No other main effects or interactions approach significance (all p>.1).

To further investigate the interaction of lexical category and hemisphere, t-tests were conducted comparing the mean amplitude of response for all noun tokens to all verb tokens (collapsed over lexical status) over each hemisphere. Verbs produced a reliably lower mean amplitude than nouns over left hemisphere sites (M_noun_=0.415; M_verb_=−0.008; t(16)=2.94, p=0.01, Cohen’s d=.471), but not over right hemisphere sites (M_noun_=0.847; M_verb_=−0.889; t(16)=0.286, p=0.779, Cohen’s d=0.058).

To further examine the effect of lexical status on these category-based differences, t-tests were conducted over left hemisphere sites. Real nouns produce greater mean amplitude in this first positive segment than real verbs do (M_noun_=0.521; M_verb_=−0.01; t(16)=2.641, p=0.018, Cohen’s d=0.694). No difference in mean amplitude is found for nonsense words over the same sensors (M_noun_=0.31; M_verb_=−0.004; t(16)=1.35, p=0.196, Cohen’s d=0.336).

#### 3.2.2 N1 Amplitude

Analysis of the initial negative peak indicates no significant main effects of any factor, but does find a marginal effect of lexical category (F(1,16)=3.58, p=.077, partial η^2^=.183), indicating that verbs produce greater negativity than nouns do. The ANOVA also shows a significant lexical category by hemisphere interaction (F(1, 16)= 7.17, p=.017, partial η^2^=.309) and a marginal interaction of lexical status and lexical category (F(1, 16)=4.072, p=.061, partial η^2^=.203). No other main effects or interactions approach significance (all p>.1).

To investigate the interactions of lexical category and hemisphere in this time period, t-tests were performed comparing mean amplitude for nouns to that for verbs, collapsed over lexical status, for each hemisphere. Verbs produce significantly greater negativity than nouns over left hemisphere sites (M_noun_=−0.261; M_verb_=−0.918; t(16)=3.629, p=0.002, Cohen’s d=0.653), but not over right hemisphere sites (M_noun_=−0.169; M_verb_=0.041; t(16)=.959, p=0.352, Cohen’s d=0.138), indicating that this effect is confined to the left hemisphere.

The interaction of lexical status and lexical category was also further examined. Collapsed across hemispheres, verbs produce significantly greater negativity than nouns do when the words are real (M_noun_=−0.137; M_verb_=−0.566; t(16)=3.11, p=0.007, Cohen’s d=0.397), but not when words are nonsense (M_noun_=−0.293; M_verb_=−0.312; t(16)=0.112, p=0.912, Cohen’s d=0.019). This shows that differences in response to noun and verb tokens require that the words have a lexical status.

#### 3.2.3 Slow, Late Component Amplitude

Mean amplitude of the longer, later negative component shows no significant main effect of any factor (all p>.1), a significant lexical category by lexical status interaction (F(1,16)=6.8, p=.019, partial η^2^=.298) and a marginal lexical category by hemisphere interaction (F(1, 16)=3.74, p=.071, partial η^2^=.19). No other interactions approached significance (all p>.25). As with the previous component, verbs elicit greater average negativity than nouns in the left hemisphere (M_noun_=−0.159; M_verb_=−0.663; t(16)=2.9, p=0.01, Cohen’s d=0.213), but not the right hemisphere (M_noun_=−0.709; M_verb_=−0.577; t(16)=0.554, p=0.587, Cohen’s d=0.053) when collapsed over lexical status. Likewise, when collapsed across hemisphere, real verbs evoke greater negativity than real nouns (M_noun_=−0.274; M_verb_=−0.771; t(16)=2.363, p=0.031, Cohen’s d=0.212), an effect that does not hold for nonsense words (M_noun_=−0.593; M_verb_=−0.469; t(16)=0.958, p=0.352, Cohen’s d=0.063). Because the interactions do not change between this time frame and the first negative segment, these effects likely arose during that first negative segment and are merely continuations of those patterns, making this segment of the data not a component distinct from the previous one.

## 4. Discussion

This study asks whether adult native speakers of English distinguish between noun and verb uses of noun/verb homophones when syntactic information is not available. The findings show that ERP responses to word tokens reliably indicate whether the token was a noun or a verb. In other words, adults neurally distinguish noun and verb uses of homophones based on pronunciation alone. Because no such effects are found for nonsense words extracted from exactly the same sentence contexts, these findings suggest that adults encode the durational differences between noun and verb tokens of homophones as part of the word’s identity.

Prior work has shown that adults reliably produce distinctions between noun and verb uses of homophones (Conwell & Barta, under revision; Sorensen, et al., 1978), but whether those distinctions arise as a property of the words themselves or as a by-product of the relationship between syntax and prosody has remained an open question. Additionally, the availability of these differences to adult listeners has been unaddressed. The research presented here resolves the question of availability and suggests that the distinctions could be encoded in the lexical representation. Taken together with research on infants’ perception of noun/verb homophones, these findings indicate that homophone distinctions are learned during the acquisition process and maintained across development.

The findings of this study are consistent with previous ERP results regarding noun/verb ambiguous words. Specifically, Federmeier and colleagues (2000) also find an effect of lexical category (for both ambiguous and unambiguous words) in a negative-going component, although that component was seen bilaterally and more posterior than the effect described here. Federmeier and colleagues presented their stimuli visually. When auditory tokens of ambiguous words were presented in disambiguating contexts by Brown and colleagues (1973; 1976; 1979), differences emerged in a negative-going component around 150 ms post-onset, an effect similar to that found in the present study. Additionally, auditory presentation of unambiguous nouns and verbs elicits a difference in a negative component between 150–200 ms post-onset in both children and adults (Molfese, et al., 1996; Tan & Molfese, 2009). All of these effects occur at left, frontal sites. Taken together with the present work, these findings suggest that auditory noun and verb tokens are differentiated neurally. For unambiguous words, this effect does not require syntactic information about lexical category because that information is already encoded as part of the lexical representation. For ambiguous words, the noun and verb tokens must be distinguished, either by the syntax (Brown, et al., 1973; 1976) or by acoustic properties indicative of lexical category, as shown in the current study.

These findings offer a new perspective on the representation of category ambiguous words in English. Like infants, adults are able to distinguish between noun and verb uses of the same word extracted from a sentence context. The only possible cues to lexical category in these tokens are acoustic/phonetic differences, such as changes in the duration of the vowel. Because adults not only make these distinctions but also appear to link the differences to specific categories, they may have, as children, learned to associate token and vowel duration with the lexical category of ambiguous words. This suggests that, like other homophones, reliable phonetic distinctions are associated with each use of the word and that these distinctions may, in fact, be part of the lemma itself (cf., Gahl, 2008, regarding homophones more broadly).

Previous work has suggested that the acoustic distinctions between noun and verb uses of the “same” word arise from the prosodic patterns associated with the sentence positions in which nouns and verbs typically appear (Conwell & Morgan, 2012; Sorensen, et al., 1978). This research does not contradict that possibility. Rather, it supports an account by which the prosodic effects become linked to the lemma itself. The idea that non-segmental information can be attached to lexical representations has significant empirical support. Lexical recognition can be affected by speaker identity (Nygaard & Pisoni, 1998), emotion (Nygaard, et al., 2002), speaking rate (Bradlow, Nygaard & Pisoni, 1999) and environmental sounds (Pufahl & Samuel, 2014). Because lexical representations can be influenced by information that is not relevant to lexical identity or function (such as speaker identity), grammatically-relevant supra-segmental information might also influence word learning and, over time, that information could become attached to the lexical representation. The data presented in this article indicate that prosodic information can be encoded as part of the lexical representation and, moreover, that it might be linked to grammatical function. Such richness of representation could facilitate both learning and processing of noun/verb homophones.

In positing supra-segmental information as part of the lexical representation, an additional question arises about the processing of homophones in their written form.^2^ Indeed, much of the research on neural correlates of noun/verb homophone processing uses visual, rather than auditory, presentation (e.g., Federmeier, et al., 2000; Pulvermüller, et al., 1999) and adds syntactic context to disambiguate meaning. The data presented in this article, along with previous work on the role of supra-segmental information in disambiguating noun/verb homophones, would imply that written homophones are more ambiguous than their spoken counterparts because they lack the acoustic cues to disambiguation that are present in speech. In this case, processing a written homophone might be similar to processing temporary ambiguity in language more generally. A reader would need to incorporate such cues as syntax, semantic context and lemma frequency to resolve that ambiguity.

An alternative interpretation that must be considered is the possibility that all of the effects reported in this article may be a result of the acoustic distinctions in the stimuli without reference to the lexical representation. There is no question that these effects are based on the acoustic properties of the words, but whether those acoustic properties are inherently connected to the lemma is a more difficult question to address. The physical properties of the stimuli, however, do not necessarily predict the pattern seen in the ERP response. In particular, if the difference between real nouns and real verbs is an artifact of noun uses having longer token and vowel duration, one would expect the nonsense words to produce an ERP that is overall greater than the real words, as the nonsense words were, on average, longer than the real word tokens. This does not eliminate the possibility that the neural response is driven entirely by the physical properties of the stimuli, but suggests that such an account would need to explain why the interactions of lexical category and lexical status found in the ERPs are not found in the acoustic analyses of the stimuli. Because the interaction of lexical category and lexical status in the ERP is not present in the acoustic properties of the stimuli, it is unlikely that acoustics alone can account for the interaction found in the neural response.

If, as the current research implies, noun and verb representations of ambiguous words differ in acoustic as well as in grammatical properties, a number of new questions open up. For example, how might these acoustic properties interact with grammatical information in sentence processing? Do conflicts between the two types of cues slow processing? Will children (or adults) use these cues in a word-learning task? Although these questions are outside the scope of the current research, the burgeoning evidence that lexical representations of homophones may link pronunciation with meaning suggests that these questions will need to be addressed to more completely understand the structure of the lexicon.

The data presented in this article indicate that adult native speakers of English neurally differentiate noun and verb uses of homophones even when the only cues to lexical category are acoustic in nature. Because infants also show the ability to distinguish between such uses, this supports the hypothesis that this ability does not weaken during development in the same way that non-native phoneme sensitivity does. Rather, these distinctions are maintained across the lifespan to support learning and processing of noun/verb ambiguity in English.

## Highlights

- Noun/verb homophones contain different acoustic information depending on category
- We recorded ERP responses to auditory noun/verb homophones and nonce words
- Nouns and verbs elicit difference in ERP as early as 100 ms after stimulus onset
- Findings suggest that acoustic differences could be part of lexical representations

## Footnotes

1 This is an unusually high participant attrition rate for neurologically typical adults. The reason for such a high rate of participant loss is unknown, although the small number of usable trials among the excluded participants suggests that they were not following the instructions to minimize movement.

2 I thank an anonymous reviewer for raising this point.
